# Effects of Dried Pumpkin Powder on the Gel Properties and Protein Structural Characteristics of *Trichiurus haumela* Surimi

**DOI:** 10.3390/foods15183348

**Published:** 2026-09-21

**Authors:** Fei Feng, Shan Yu, Yuchun Xu, Jie Pang, Chunhua Wu

**Affiliations:** 1 College of Culinary and Food Science Engineering, Sichuan Tourism University, Chengdu 610100, China; feng15892004@163.com; 2 College of Food Science, Fujian Agriculture and Forestry University, Fuzhou 350002, China; 3 Key Laboratory of Plant Resource Conservation and Germplasm Innovation in Mountainous Region (Ministry of Education), College of Life Science, Guizhou University, Guiyang 550025, China

**Keywords:** dried pumpkin powder, hairtail surimi, protein secondary, structure, microstructure

## Abstract

The poor gel-forming ability of myofibrillar proteins in hairtail (*Trichiurus haumela*) surimi limits its application in high-quality surimi products. This study investigated the effects of dried pumpkin powder (DPP) addition (0, 0.4%, 0.75%, 1.1%, 1.5%, and 1.85%, *w*/*w*) on the gel properties, protein conformation, and microstructure of frozen hairtail surimi gels. The results revealed a nonlinear, dose-dependent effect on gel quality. At 1.1% addition, gel hardness and springiness reached maximum values of 313.69 ± 2.54 g and 0.86 ± 0.004, respectively, while water-holding capacity (WHC) significantly increased by 11.21% compared with the control (*p* < 0.05). Fourier-transform infrared spectroscopy demonstrated a marked reduction in α-helix content with a concomitant increase in random coil content, indicating changes in protein secondary structure that may favor interactions within the protein network. Scanning electron microscopy further revealed that moderate supplementation filled network voids and produced a denser, more homogeneous microstructure, whereas excessive addition (≥1.5%) led to fiber aggregation and structural deterioration. Collectively, moderate pumpkin powder addition—specifically at 1.1%—improves hairtail surimi gel quality, and these improvements may be associated with changes in protein secondary structure and microstructural organization, offering a practical strategy for developing fiber-enriched surimi products.

## 1. Introduction

Hairtail (*Trichiurus haumela*) is one of the most economically important marine fish species in China, characterized by high seasonal catches and substantial processing volume. Beyond fresh consumption, a large proportion of hairtail is processed into surimi-based products such as fish balls, fish sausages, and imitation crab sticks [1,2]. The quality of these products critically depends on the gel-forming capacity of myofibrillar proteins, which governs textural attributes and water-holding capacity (WHC). However, the inherently poor gelation properties of hairtail myofibrillar proteins limit the formation of a strong and stable gel network, resulting in suboptimal product quality and restricting the industrial exploitation of this resource [3]. Consequently, improving the gelation performance of hairtail surimi remains a major challenge for its value-added utilization.

The incorporation of plant-derived ingredients has emerged as an effective and practical strategy for improving the gel quality of surimi. Among various modifiers, plant-derived polysaccharides and dietary fibers have attracted increasing attention because of their natural origin, cost-effectiveness, and desirable nutritional and techno-functional attributes. Numerous studies have demonstrated that appropriate supplementation with these biopolymers can enhance surimi gelation through multiple mechanisms, including filler effects that reinforce the gel matrix, molecular interactions with myofibrillar proteins that promote network formation, and enhanced water retention that improves overall product quality [4,5,6]. For example, plant-based dietary fibers derived from citrus, oat, wheat, and bamboo shoot have been shown to strengthen intermolecular interactions, facilitate protein structural rearrangement, and promote the formation of more compact and homogeneous gel networks in various surimi systems [7,8,9,10]. Despite these promising results, many of the reported approaches rely on complex processing techniques or specially formulated ingredients, which may escalate production costs and hinder large-scale industrial adoption. Therefore, the development of simple, natural, and economically viable plant-derived additives for improving surimi gelation remains a priority.

Pumpkin (*Cucurbita moschata*) is a widely cultivated and nutritionally rich plant resource, containing substantial amounts of dietary fiber, polysaccharides, proteins, carotenoids, and other bioactive components [11,12]. Dried pumpkin powder (DPP), mainly composed of cellulose, hemicellulose, pectin, and lignin, possesses a porous microstructure, high hydration capacity, and excellent water-binding properties [13,14]. These physicochemical characteristics position DPP as a promising functional ingredient for regulating food texture and gel properties. Previous studies have shown that DPP can reduce cooking loss and improve the textural properties of emulsified meat products [15]. However, its application in surimi products has received little attention, and the effects of pumpkin powder on the gelation behavior of surimi and the underlying mechanisms remain largely unexplored.

Based on the above background, the present study systematically investigated the effects of different addition levels of DPP (0.4%, 0.75%, 1.1%, 1.5%, and 1.85%, *w*/*w*) on the WHC, cooking loss, textural properties, sensory quality, protein secondary structure, and microstructure of the surimi gel. The primary objectives were to identify the optimal addition level of DPP and to elucidate the potential mechanisms by which it improves surimi gel quality. The findings are expected to provide a theoretical basis and technical reference for the high-value utilization of DPP in aquatic food processing and for the development of premium hairtail surimi products.

## 2. Materials and Methods

### 2.1. Materials

Frozen hairtail (*Trichiurus haumela*) surimi was supplied by Fujian Shengtian Food Co., Ltd. (Fuzhou, China) and stored at −80 °C until use. Prior to experiments, the frozen surimi was thawed overnight at 4 °C, and its moisture content was 78.0%. Pumpkin (*Cucurbita moschata*, Xiangyu cultivar) was obtained from a local agricultural market in Fujian, Fuzhou, China. Dried pumpkin powder (DPP) was prepared from dried pumpkin flesh by grinding, followed by sieving through a 100-mesh sieve to obtain a relatively uniform powder.

### 2.2. Methods

#### 2.2.1. Preparation of Surimi Gels

DPP was incorporated into thawed hairtail (*Trichiurus haumela*) surimi at mass fractions of 0 (control), 0.4, 0.75, 1.1, 1.5, and 1.85% (*w*/*w*, based on the wet weight of the thawed surimi). The mixtures were chopped for 5 min using a bowl cutter (JJ-2BX, Fangke Instrument Co., Ltd., Changzhou, China) until a homogeneous paste was obtained. During chopping, the temperature of the surimi gels was maintained below 10 °C to minimize protein denaturation. The resulting surimi gels were carefully transferred into 10 mL glass beakers, and entrapped air bubbles were removed prior to sealing. Gelation was performed using a two-step heating procedure consisting of incubation at 40 °C for 60 min, followed by heating at 90 °C for 35 min in a thermostatic water bath (HH-1, Jintan Liangyou Instrument Co., Ltd., Changzhou, China). After thermal treatment, the gels were immediately cooled under running tap water to room temperature and subsequently stored at 4 °C overnight before further analyses. No additional NaCl, water, cryoprotectants, or other ingredients were added during the preparation of the surimi gels, except for the designated amount of dried pumpkin powder (DPP).

#### 2.2.2. Texture Profile Analysis

Texture profile analysis (TPA) of surimi gels was performed using a texture analyzer (TA. XT plus, Stable Micro Systems Ltd., Godalming, UK) equipped with a P/36 R cylindrical probe, following the method of ZHANG et al. [16] with minor modifications. Surimi gel samples were cut into cylinders of 20 mm in height. The test conditions were as follows: pre-test and post-test speeds of 2.0 mm/s, test speed of 1.0 mm/s, compression distance of 6 mm (approximately 30% strain), and trigger force of 5 g. Hardness and springiness were recorded as key texture parameters. Each sample was measured in six replicates.

#### 2.2.3. Water-Holding Capacity (WHC) and Cooking Loss

The water-holding capacity (WHC) of surimi gels was determined according to the method described by Xiong et al. with slight modifications [17]. Approximately 10.0 g of surimi gel was weighed (m_1_), wrapped completely with filter paper, and centrifuged at 4000× *g* for 15 min at 4 °C using a refrigerated centrifuge (H2-16KR, Kecheng Instrument Equipment Co., Ltd., Changsha, China). After centrifugation, the filter paper was carefully removed, and the remaining gel was reweighed (m_2_). WHC is calculated using the following formula:

$$\mathrm{WHC}\left(\%\right)\;=\;\frac{m_{2}}{m_{1}}\;\times \;100$$

Cooking loss was measured following the method of Lv et al. [18] with slight modifications. Approximately 10.0 g of surimi paste was weighed (w_1_), transferred into a heat-resistant polyethylene bag, and vacuum-sealed. The samples were subjected to a two-step heating process consisting of incubation at 40 °C for 60 min, followed by heating at 90 °C for 35 min in a thermostatic water bath (HH-1, Jintan Liangyou Instrument Co., Ltd., Changzhou, China). After thermal treatment, the samples were immediately cooled under running tap water to room temperature. Surface moisture was carefully removed using absorbent paper before the samples were reweighed (w_2_). Cooking loss was calculated as follows:

$$\mathrm{Cooking}\;\mathrm{loss}\;\left(\%\right)\;=\;\frac{w_{1}-w_{2}}{w_{1}}\;\times \;100$$

#### 2.2.4. Dynamic Rheological Properties

Dynamic rheological measurements of uncooked surimi pastes were conducted using a rheometer (MCR301, Anton Paar GmbH, Graz, Austria) equipped with a parallel plate geometry (PP50, diameter 50 mm), following the method of Xiong et al. [15] with slight modifications. The gap between plates was set to 1 mm, and the strain amplitude was maintained at 1.5% within the linear viscoelastic region. The samples were subjected to a temperature sweep from 25 to 90 °C at a heating rate of 5 °C/min and a fixed frequency of 1 Hz. The storage modulus (G′) and loss modulus (G″) were recorded as functions of temperature. Each measurement was performed in triplicate.

#### 2.2.5. Whiteness Measurement

The whiteness of surimi gels was measured using a color difference meter (CS-200, Hangzhou CHNSpec Technology Co., Ltd., Hangzhou, China), following the method described by Xiong et al. [19] with slight modifications. Before measurement, the instrument was calibrated using a standard white calibration plate according to the manufacturer’s instructions. Surimi gels were cut into slices approximately 25 mm in thickness, and the color parameters L* (lightness), a* (redness/greenness), and b* (yellowness/blueness) were recorded. Whiteness (W) was calculated according to the following formula:

$$W\;=\;100\;-\sqrt{{\left(100\;-\;L^{*}\right)}^{2}\;+\;{a^{*}}^{2}\;+\;{b^{*}}^{2}}$$

Each sample was measured in six replicates.

#### 2.2.6. Sensory Evaluation

Sensory evaluation was conducted by a trained panel consisting of 12 assessors (6 males and 6 females; 20–25 years old). All panelists had received professional training in sensory evaluation prior to the formal assessment and were familiar with the evaluation procedure and scoring criteria. Each surimi gel sample was coded with a random three-digit number and presented to the panelists in randomized order. The sensory quality of the samples was evaluated using a 0–100 scoring scale based on four attributes: color, odor, taste, and texture. The weighting coefficients assigned to color, odor, taste, and texture were 20%, 25%, 25%, and 30%, respectively [20]. A higher score indicated better sensory quality for the corresponding attribute. The total sensory score was calculated as follows:

$$Total\;sensory\;score=\left(color\;score\times 0.20\right)+\left(odor\;score\times 0.25\right)\;+(taste\;score\times 0.25)+(texture\;score\times 0.30)$$

The final sensory score for each sample was calculated as the mean score obtained from the 12 panelists.

#### 2.2.7. Protein Secondary Structure Analysis

The secondary structure of proteins in surimi gels was analyzed using Fourier-transform infrared (FT-IR) spectroscopy (Nicolet iS50, Thermo Fisher Scientific Inc., Waltham, MA, USA), following the method of Wei et al. [21] with modifications. Freeze-dried surimi gel samples were ground with potassium bromide (KBr) in an agate mortar until no visible particles remained, and the mixture was compressed into transparent pellets. FT-IR spectra were recorded in the wavenumber range of 4000~400 cm^−1^ with a resolution of 4 cm^−1^ and 32 scans per sample. The amide I region (1700~1600 cm^−1^) was subjected to Fourier self-deconvolution and iterative curve-fitting using OMNIC software (version 32, Thermo Fisher Scientific) and PeakFit software (version 4.12, Systat Software Inc., San Jose, CA, USA), respectively. The relative contents of ɑ-helices, β-sheets, β-turns, and random coils were calculated from the integrated areas of the corresponding sub-peaks [22]. Each sample was analyzed in triplicate.

#### 2.2.8. Microstructure Observation

The microstructure of surimi gels was observed using scanning electron microscopy (SEM) according to the methods of Ding et al. [22] with slight modifications. Surimi gel samples were cut into thin slices (approximately 2 mm thickness) and fixed in 2.5% glutaraldehyde solution (in 0.1 M phosphate buffer, pH 7.2) for 12 h at 4 °C. After fixation, the samples were rinsed three times with phosphate buffer (pH 7.2) for 1 h, and subsequently dehydrated in graded ethanol solutions (30%, 50%, 70%, 80%, 95%, and 100%, *v*/*v*) for 15 min each. The dehydrated samples were then freeze-dried, sputter-coated with gold, and examined using a scanning electron microscope (Regulus 8100, Hitachi Ltd., Tokyo, Japan) at an accelerating voltage of 5 kV. The pore area and perimeter of the gel network were measured from the SEM images using Image-J software (version 1.54, National Institutes of Health, Bethesda, MD, USA). At least 10 randomly selected fields were analyzed for each sample.

### 2.3. Statistical Analysis

All treatments were independently prepared in triplicate (three independent experimental replicates, *n* = 3). For each experimental replicate, analytical measurements were performed in triplicate unless otherwise specified. The mean of the analytical replicates was used as the value for each independent experimental replicate, and the three independent experimental replicates were used for statistical analysis. Results are expressed as the mean ± standard deviation (SD). Statistical analyses were performed using IBM SPSS Statistics 22.0 (IBM Corp., Armonk, NY, USA). Differences among treatments were evaluated by one-way analysis of variance (ANOVA), followed by Duncan’s multiple range test. Differences were considered statistically significant at *p* < 0.05. Data visualization was performed using OriginPro 2021 (OriginLab Corp., Northampton, MA, USA).

## 3. Results and Discussion

### 3.1. Texture Properties of Surimi Gels

**Figure 1:**
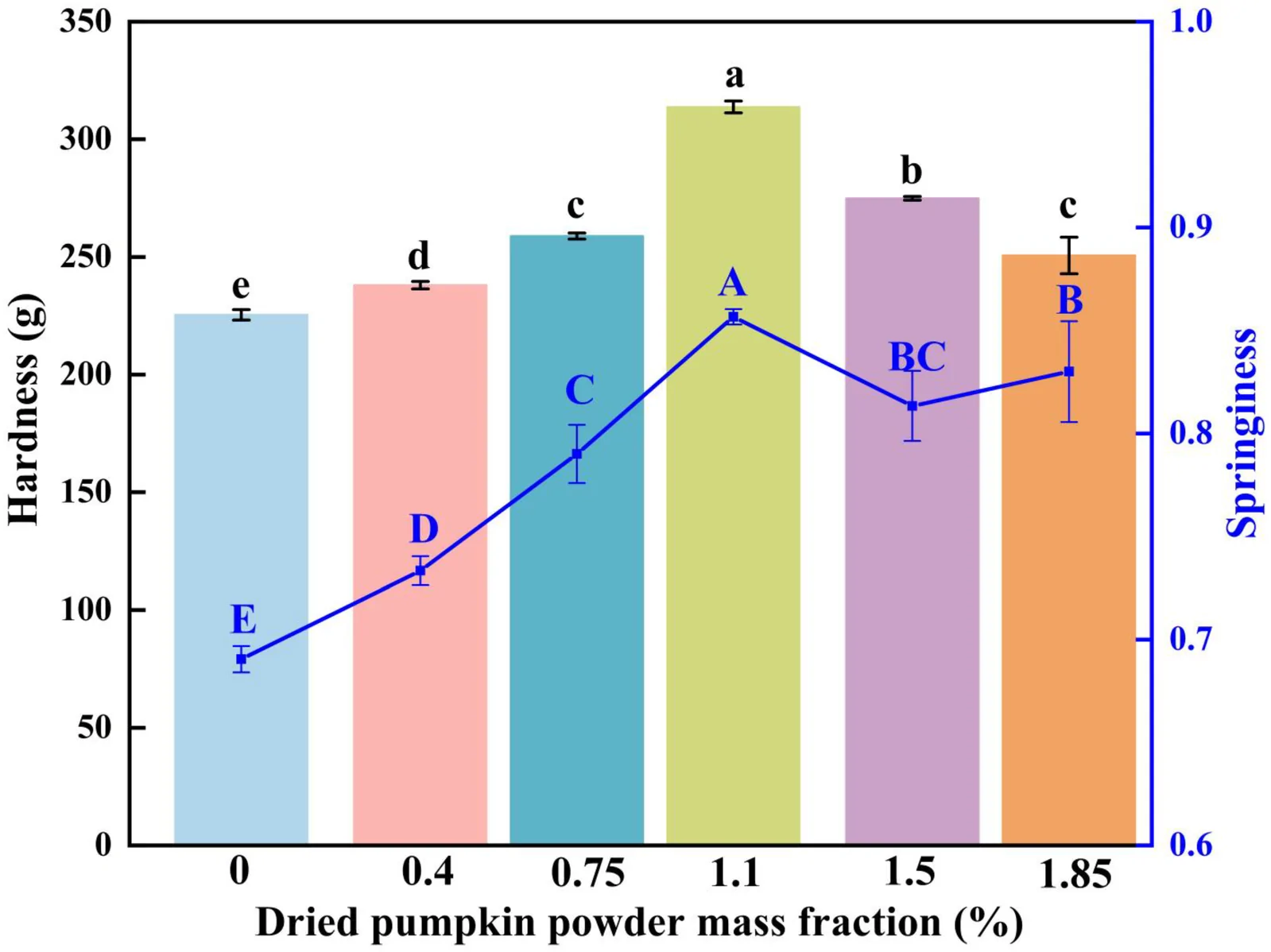
Effect of DPP content on the hardness and springiness of surimi gel. Lowercase and uppercase letters indicate significant differences in hardness and springiness, respectively (*p* < 0.05).

Textural properties are key indicators for evaluating the quality of surimi gel products. As shown in Figure 1 for DPP, with increasing DPP content, the hardness of the hairtail surimi gel exhibited a three-phase response—initially increasing, then declining—whereas springiness fluctuated in a non-monotonic manner, first increasing, then decreasing, and subsequently increasing. At a DPP addition of 1.1%, both hardness and elasticity reached their maximum values, at 313.69 ± 2.54 g and 0.86 ± 0.004, respectively; further increases in addition level led to a progressive reduction in both parameters. These results demonstrate that moderate DPP supplementation significantly improves the textural properties of surimi gels, while excessive incorporation exerts a detrimental effect.

The observed improvement in hardness and elasticity can be attributed to DPP. During heating, DPP particles absorb water and swell, occupying the voids within the protein gel network, thereby increasing the structural density and resistance to deformation of the system [23]. At the optimal addition level of 1.1%, DPP particles were presumably well-dispersed and effectively integrated with the protein matrix, forming a well-structured composite network that simultaneously enhanced both hardness and springiness. This phenomenon is consistent with the findings of Jia et al. [18] and Huang et al. [24], who reported that appropriate amounts of dietary fiber, acting as an “active filler”, reinforced the gel network of surimi products. Conversely, an increased proportion of non-protein material within the system may reduce effective protein–protein contacts and create local discontinuities in the gel matrix, resulting in lower resistance to deformation and reduced springiness [24]. Collectively, these results confirm that the addition of pumpkin powder at an optimal level (1.1%) holds considerable potential for improving the texture of hairtail surimi gels.

### 3.2. Water-Holding Capacity of Surimi Gels

**Figure 2:**
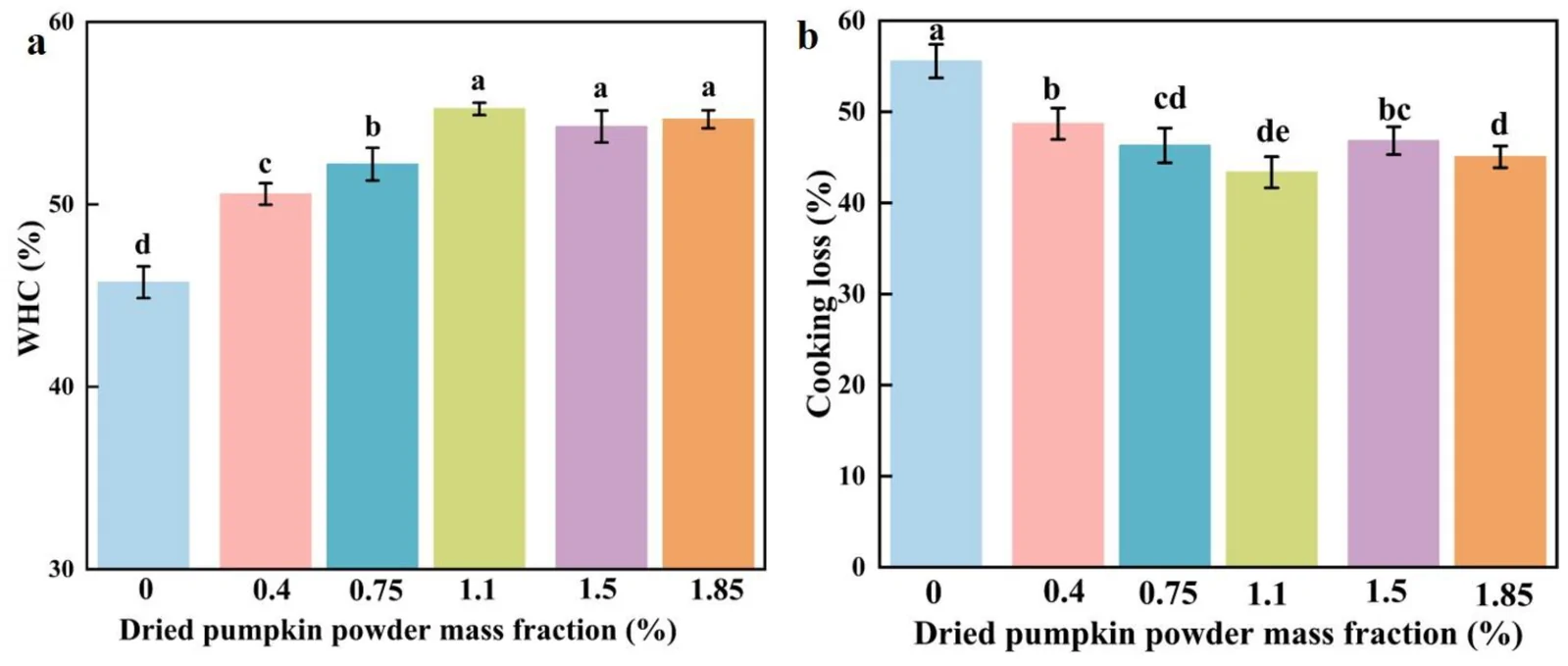
Effect of DPP addition on (**a**) WHC and (**b**) cooking loss of surimi gel. Different lowercase letters indicate significant differences (*n* = 3, *p* < 0.05). Values sharing the same letter are not significantly different (*p* > 0.05).

WHC and cooking loss are two mutually corroborating indicators for evaluating the water retention performance of surimi gel. WHC reflects the ability of the gel network to retain water, while cooking loss characterizes the extent of juice loss during thermal processing; together, they determine the tenderness, juiciness, and economic yield of surimi products. As shown in Figure 2a, with increasing DPP addition, the WHC of hairtail surimi gel first increased and then decreased. At an addition level of 1.1%, WHC reached a maximum of 57.25%, corresponding to an increase of 11.21 percentage points, or approximately 24.3%, relative to the control group (46.04%, *p* < 0.05). Further increases to 1.5% and 1.85% resulted in a progressive decline in WHC. As shown in Figure 2b, the cooking loss rate exhibited a trend exactly opposite to that of WHC—first decreasing and then increasing—with the lowest value observed at the 1.1% addition level. These results collectively indicate that 1.1% DPP optimizes the water retention performance of surimi gels.

The mechanism by which DPP improves the WHC of surimi gels can be explained from both “chemical water absorption” and “physical water retention” perspectives. On the one hand, the molecular surface of DPP is densely populated with hydrophilic groups such as hydroxyl groups, which can bind to free water in the system via hydrogen bonding, forming a layer of bound water that is resistant to loss during centrifugation and heating. On the other hand, DPP particles absorb water and swell during heating, filling the interstitial voids within the protein gel network and rendering the structure more compact and uniform, thereby effectively physically encapsulating water through physical entrapment and capillary forces [25]. However, when the addition level exceeds 1.1%, the excess dietary fiber tends to aggregate within the system, disrupting the continuity and uniformity of the protein network and leading to the formation of larger pores, which in turn compromises water retention capacity. Collectively, these findings demonstrate that moderate pumpkin powder supplementation significantly enhances WHC and reduces cooking loss, with 1.1% as the optimal addition level. A limitation of the present study is that the detailed compositional and hydration-related properties of DPP, including dietary fiber fractions and water-binding characteristics, were not determined. Further characterization of these properties would help clarify their contributions to the observed effects on surimi gel quality.

### 3.3. Dynamic Rheological Properties of Surimi Gels

**Figure 3:**
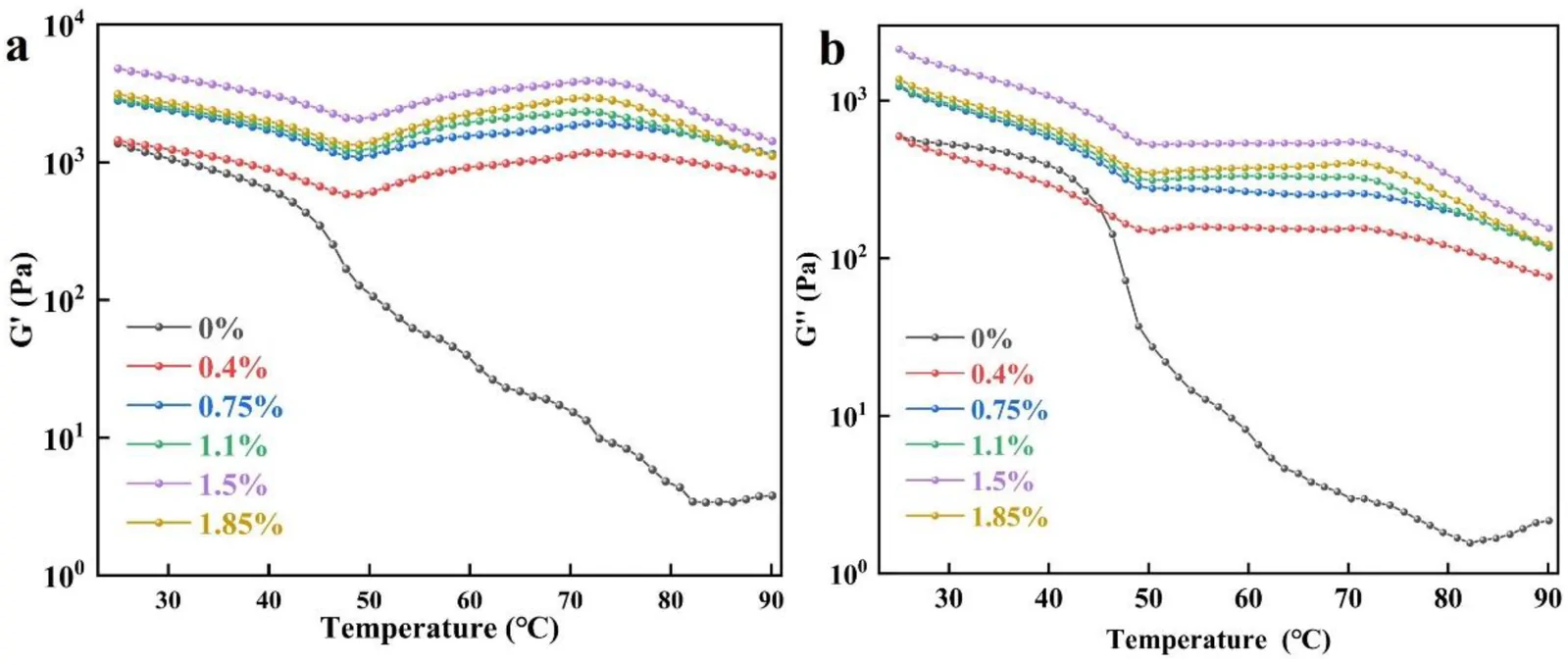
Effect of DPP addition on the dynamic viscoelasticity of surimi during heating. (**a**) storage modulus, (**b**) Loss modulus.

The storage modulus (G′) and loss modulus (G″) reflect the elastic and viscous characteristics of the gel system, respectively, and serve as key rheological parameters for characterizing the evolution of the network structure during surimi gelation. Figure 3 shows the effects of different DPP addition levels on the G′ and G″ of hairtail surimi paste gel during heating.

All samples exhibited G′ values significantly greater than G″ across the entire temperature range of 25–90 °C, indicating that the hairtail surimi system was dominated by elastic responses throughout the thermal gelation process and possessed the solid-state behavior typical of a gel [8]. Except for the control group, the DPP-added groups exhibited a three-stage trend of “decrease–sharp increase–decrease” as the temperature rose. During the initial heating stage (25–40 °C), both moduli decreased slightly, which is attributed to the partial breaking of non-covalent bonds (e.g., hydrogen bonds) maintaining the myofibrillar protein structure, leading to enhanced thermal motion of protein molecules and reduced transient cross-links [26]. As the temperature rose to 50–60 °C, both G′ and G″ increased sharply, indicating that this temperature range represents a critical stage of the sol–gel transition—the myosin heads denature and expose active groups, initially forming a cross-linked network through hydrophobic interactions and disulfide bonds. Upon further heating to 72–90 °C, both moduli decreased to varying degrees, likely due to thermal unfolding of the myosin tail (light meromyosin) region at elevated temperatures, which partially disrupts established cross-links and increases the system fluidity.

At the final temperature of 90 °C, both G′ and G″ of the 1.1% DPP group were significantly higher than those in the control group and the 1.85% group, indicating that appropriate DPP addition effectively enhances the final viscoelasticity of surimi gel. Notably, G′ and G″ did not increase linearly with DPP content—the 1.5% group exhibited higher values than the group but lower than the 1.1% group, while the 1.85% group showed a further reduction. Moderate DPP incorporation may contribute to a more favorable organization of the protein matrix, whereas excessive DPP may interfere with the continuity and organization of the developing gel network. Because the chemical composition and hydration-related properties of DPP were not characterized in the present study, the specific contribution of individual DPP components to these rheological changes cannot be conclusively determined.

### 3.4. Whiteness of Surimi Gels

**Figure 4:**
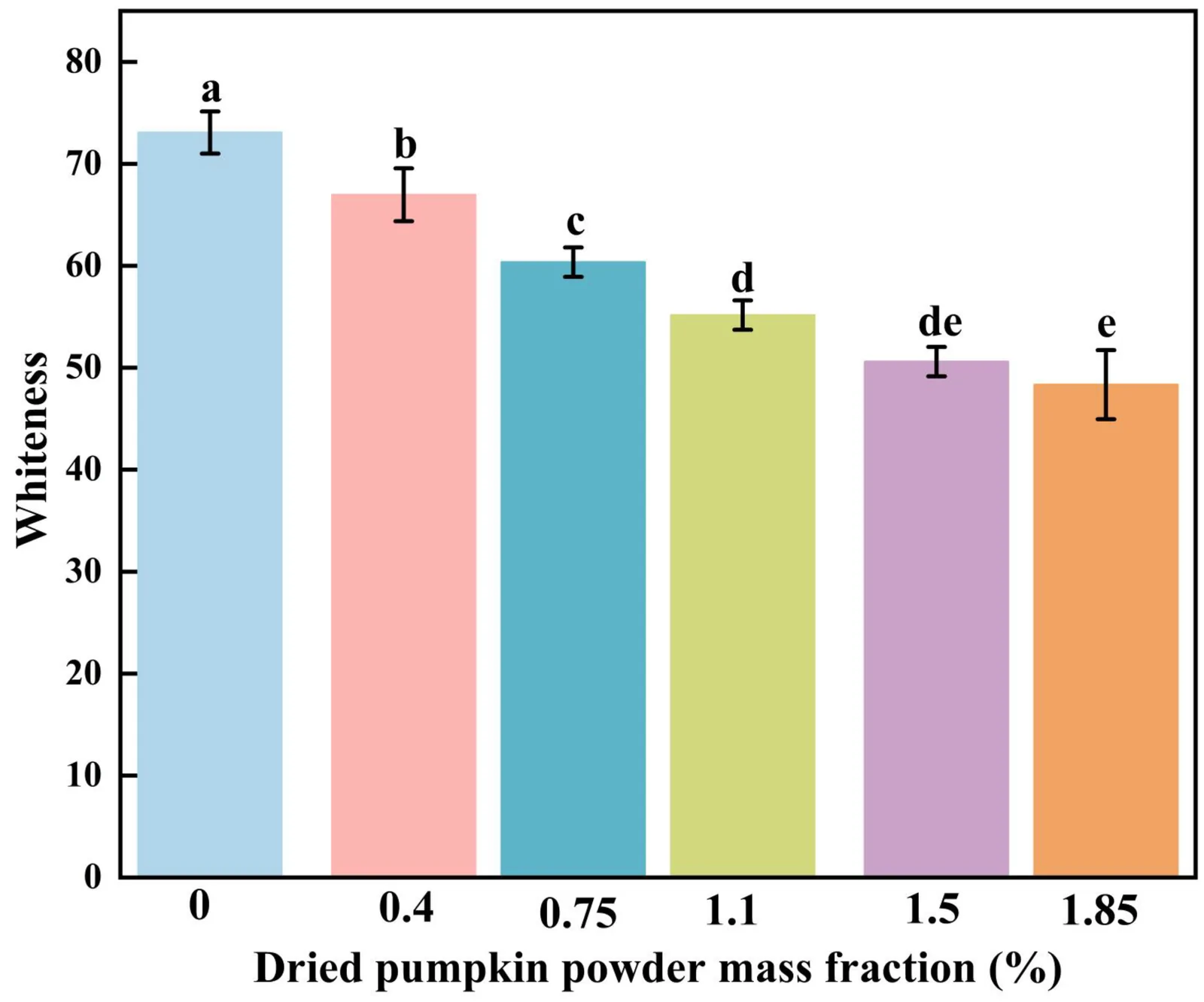
Effect of DPP addition on the whiteness of surimi gel. Different lowercase letters indicate significant differences (*p* < 0.05).

Whiteness is an important quality attribute affecting the appearance and consumer acceptance of surimi products. As shown in Figure 4, the whiteness of hairtail surimi gels decreased significantly (*p* < 0.05) with increasing DPP content. This reduction was mainly attributed to the inherent yellow-orange coloration of DPP, which is derived from its rich carotenoid content (particularly β-carotene), resulting in decreased lightness (*L*) and increased yellowness (b) of the gels. Similar declines in whiteness have been reported following the incorporation of various plant-derived dietary fibers into surimi products [8]. Despite this slight reduction in whiteness, the gel containing 1.1% DPP maintained a visually acceptable appearance while exhibiting superior gel properties, including improved texture and WHC. Given that the overall quality improvement outweighs the moderate color change, 1.1% DPP supplementation is considered practically viable for surimi processing.

### 3.5. Sensory Evaluation of Surimi Gels

**Figure 5:**
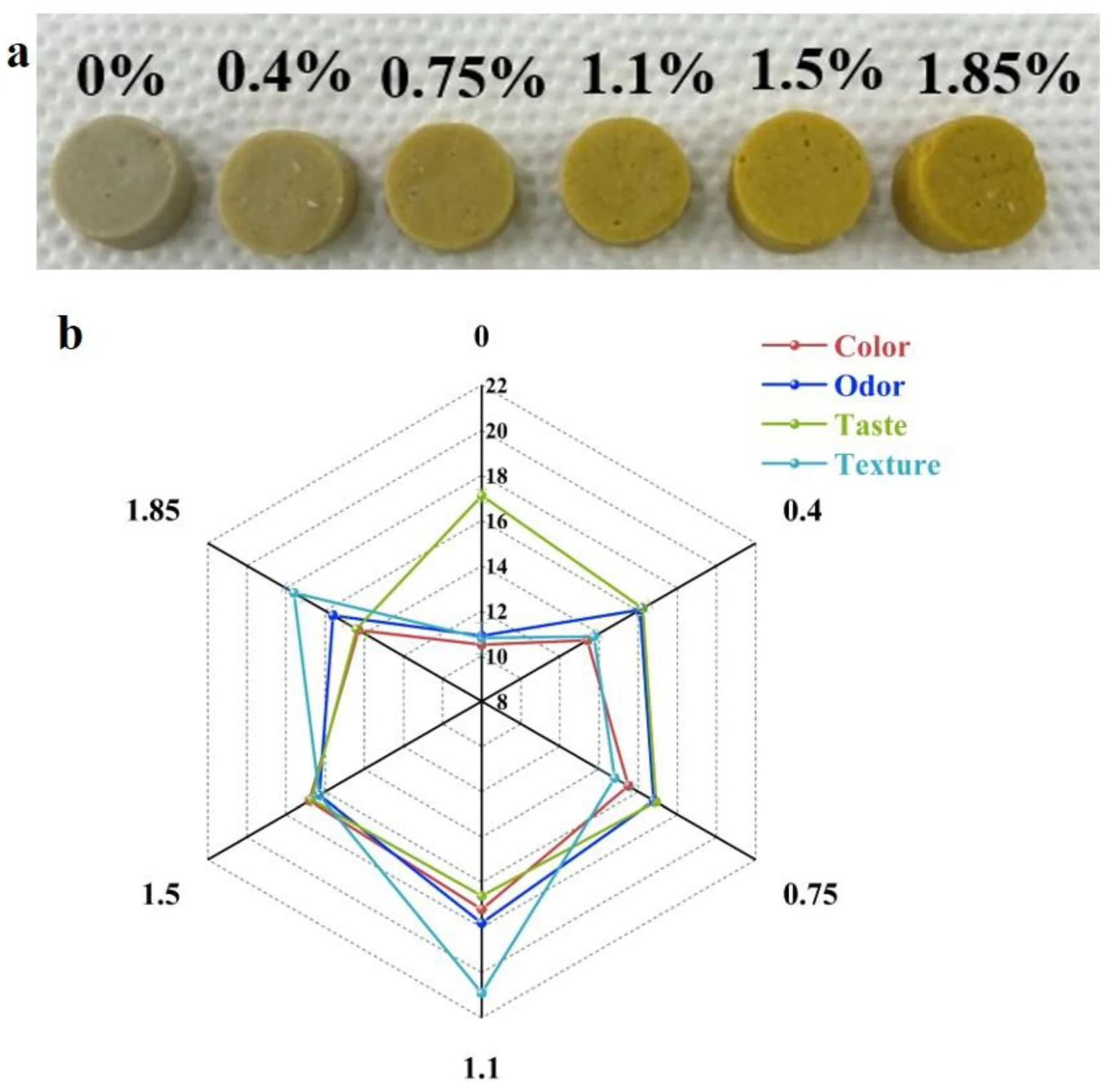
Effect of DPP addition on the (**a**) digital photographs and (**b**) sensory characteristics of surimi gel.

The sensory scores of hairtail surimi gels supplemented with different levels of DPP are summarized in Figure 5. DPP supplementation markedly affected the sensory quality of the gels. As shown in Figure 5, the color score initially increased with increasing DPP content but declined when the addition exceeded 1.1%, consistent with the whiteness results (Section 3.4) and attributable to the progressive darkening of gel appearance imparted by the natural yellow pigment of DPP. In contrast, the odor score increased gradually with increasing DPP concentration, suggesting that the characteristic aroma of DPP partially masked the fishy odor of surimi—an effect also observed with other plant-derived ingredients in surimi products, such as *Poria cocos* powder [27] and wolfberry powder [28]. The texture score followed a bell-shaped trend, increasing initially and reaching the highest value at 1.1% DPP, followed by a decline at higher supplementation levels. This result is in good agreement with the texture profile analysis (Section 3.1) and SEM observations (Section 3.7), further confirming that moderate DPP supplementation promotes the formation of a compact and well-organized gel network, whereas excessive incorporation disrupts gel structure and compromises sensory acceptability. Overall, the 1.1% DPP group achieved the highest total sensory score (72.7), demonstrating that moderate supplementation effectively enhances the overall sensory quality of hairtail surimi gels.

### 3.6. Protein Secondary Structure of Surimi Gels

**Figure 6:**
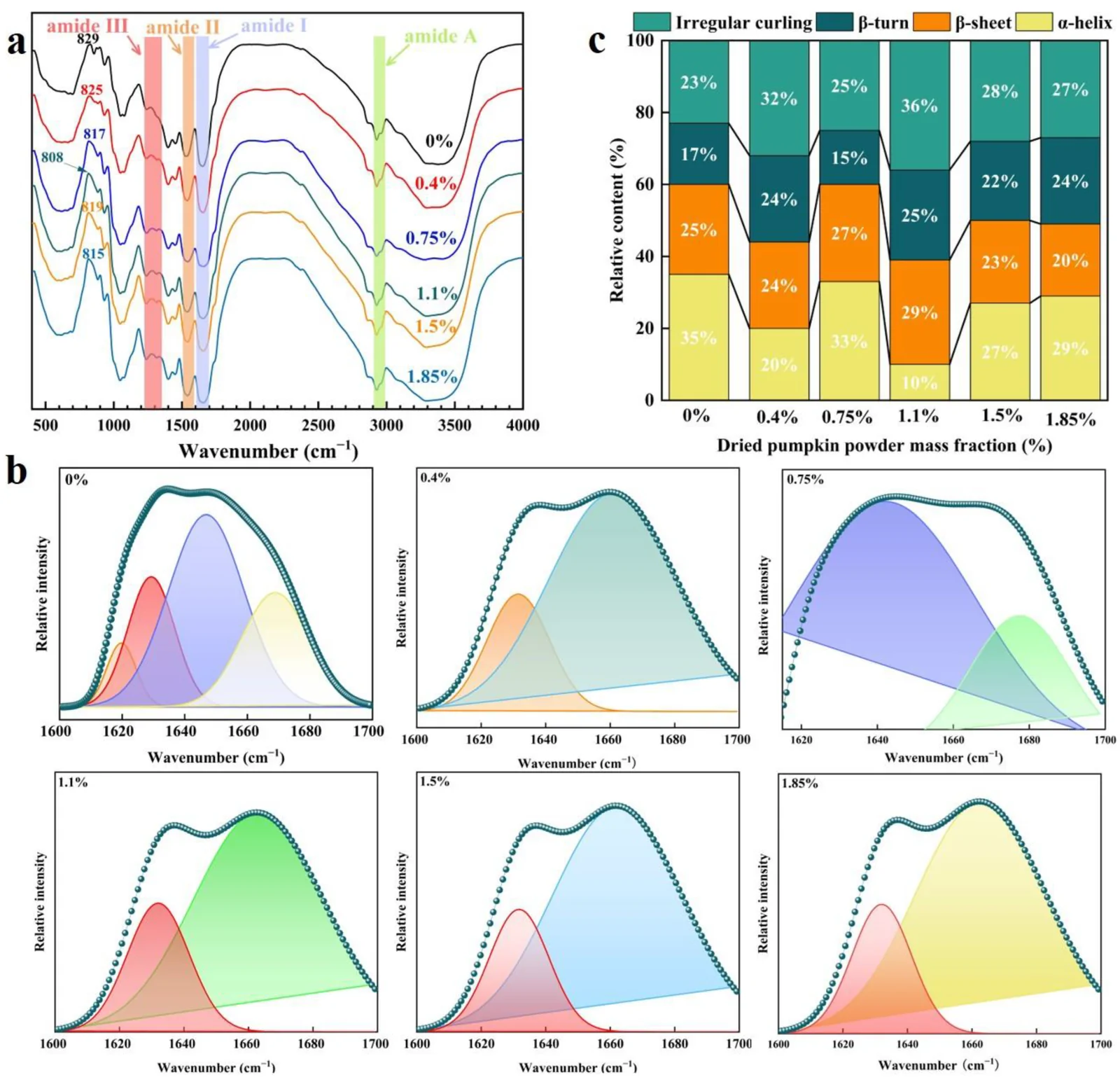
Effect of DPP addition on protein secondary structure of surimi gel: (**a**) FT-IR spectra; (**b**) iterative curve-fitting of amide I region; (**c**) relative contents of secondary structural components. The dark teal dotted line represents the experimental FTIR spectrum, and the colored filled curves represent the individual deconvoluted component bands.

FT-IR spectroscopy was employed to characterize the conformational changes in myofibrillar proteins induced by DPP. As shown in Figure 6a, all samples exhibited the characteristic absorption bands of proteins, including amide A (~3400 cm^−1^), amide I (1600–1700 cm^−1^), amide II (1500–1600 cm^−1^), and amide III (1200–1300 cm^−1^). Compared with the control, all DPP-treated samples exhibited a slight blue shift at 829 cm^−1^, which may reflect changes in the local molecular environment associated with interactions between DPP components and myofibrillar proteins [29].

To further elucidate these conformational changes, the amide I region was deconvoluted and curve-fitted to quantify the secondary structural composition (Figure 6b,c). The control group exhibited the highest α-helix content (35%). Increasing the DPP level to 1.1% markedly reduced the α-helix content to 10%, accompanied by a pronounced increase in random coil content to 36%, while β-sheet and β-turn contents reached 29% and 25%, respectively. The decrease in α-helix content together with the increase in random coil content indicates a transition from a relatively ordered secondary structure toward a less ordered and more flexible protein conformation.

These changes in protein secondary structure were associated with the observed variations in gel properties. Previous studies have suggested that a reduction in α-helical structure may accompany partial unfolding of myofibrillar proteins, which may facilitate intermolecular interactions during thermal gelation [29]. Meanwhile, the increased proportion of random coils likely imparts greater molecular flexibility, potentially favoring protein rearrangement during gel formation. Consistent with these structural changes, the 1.1% DPP group exhibited the highest hardness, springiness, and water-holding capacity, along with the most compact microstructure (Section 3.1, Section 3.2 and Section 3.7). These corresponding changes suggest that the modification of protein secondary structure may be associated with the improvement in surimi gel properties induced by DPP.

### 3.7. Microstructure of Surimi Gels

**Figure 7:**
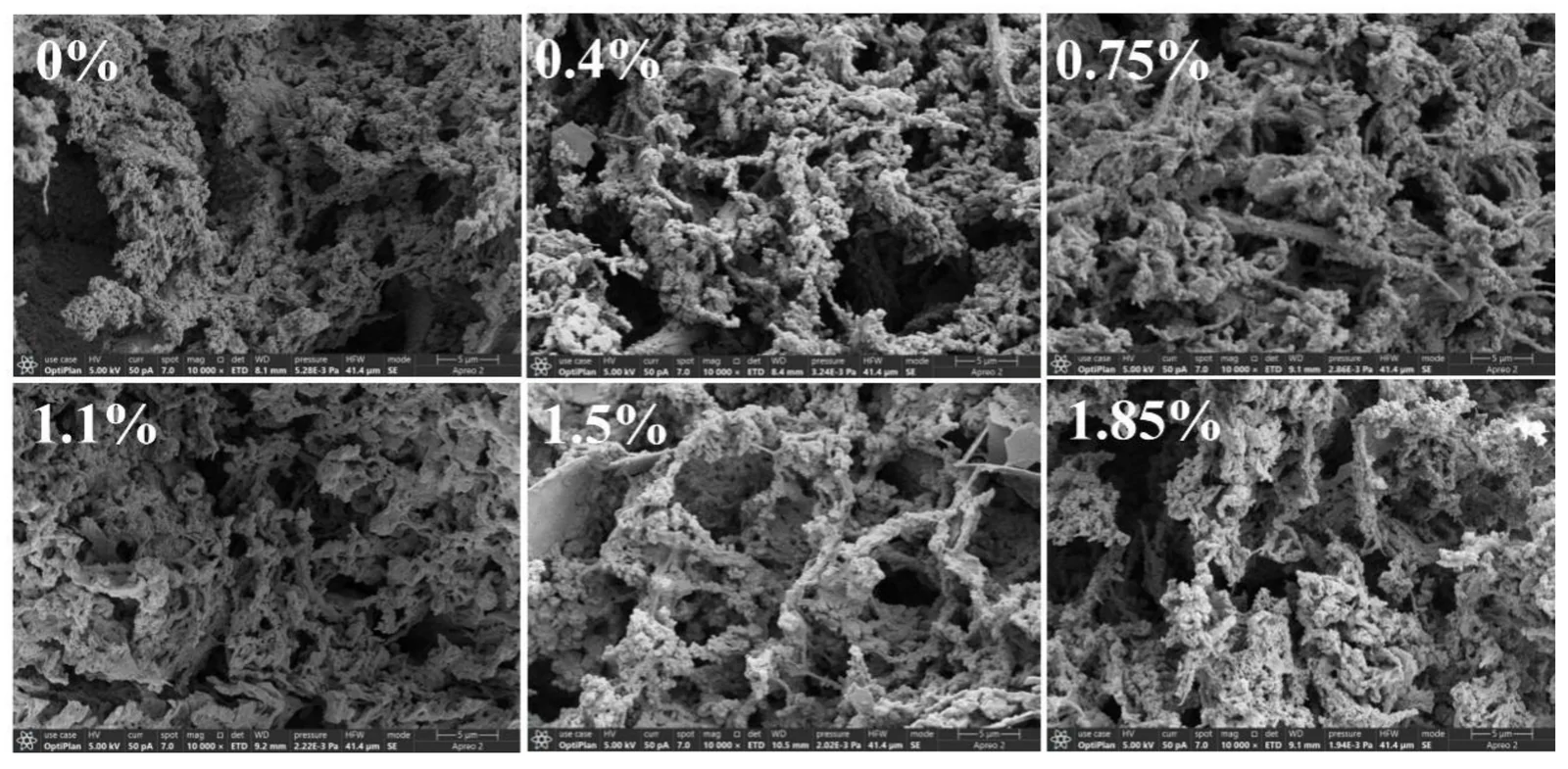
Effect of DPP addition on the microstructure of surimi gel.

SEM was employed to visualize the microstructural changes in hairtail surimi gels induced by DPP addition (Figure 7), while pore area and perimeter were quantified using ImageJ 6.0 (Silver Spring, MD, USA) (Table 1). The control gel exhibited a loose and heterogeneous network characterized by large pores and weak interconnections. Incorporation of DPP progressively refined the gel microstructure. At 0.4–0.75% supplementation, the pore structure became more homogeneous, and the protein network appeared increasingly compact. The 1.1% group exhibited the most uniform and continuous gel network, with the smallest pore area and perimeter among all groups, indicating the formation of a dense and well-organized three-dimensional structure.

The optimized microstructure was closely associated with the superior gel properties observed in the 1.1% group. A finer and more interconnected protein network generally enhances capillary water retention and reinforces the gel skeleton, thereby improving WHC, gel strength, and elasticity. These observations are consistent with the textural and water retention results (Section 3.1 and Section 3.2), suggesting that an appropriate amount of DPP is effectively associated with improved gel network organization during thermal gelation.

However, further increasing the DPP content to 1.5% and 1.85% resulted in a deterioration of the gel microstructure. Larger pores, rougher surfaces, and a more heterogeneous network were observed, indicating disruption of the continuous protein matrix. This deterioration may be attributed to excessive dietary fiber exceeding the accommodation capacity of the protein network and may be related to the uneven distribution [30,31]. Such structural heterogeneity could interfere with protein hydration and intermolecular interactions during gelation, resulting in a less integrated network with increased structural defects [31]. Consequently, excessive fiber incorporation adversely affected gel quality. Overall, these results demonstrate that moderate DPP supplementation improves surimi gel properties by optimizing the gel network architecture, whereas excessive addition induces structural heterogeneity that compromises gel formation.

### 3.8. Summary of PDF-Mediated Improvement of Trichiurus haumela Surimi Gel Quality

**Figure 8:**
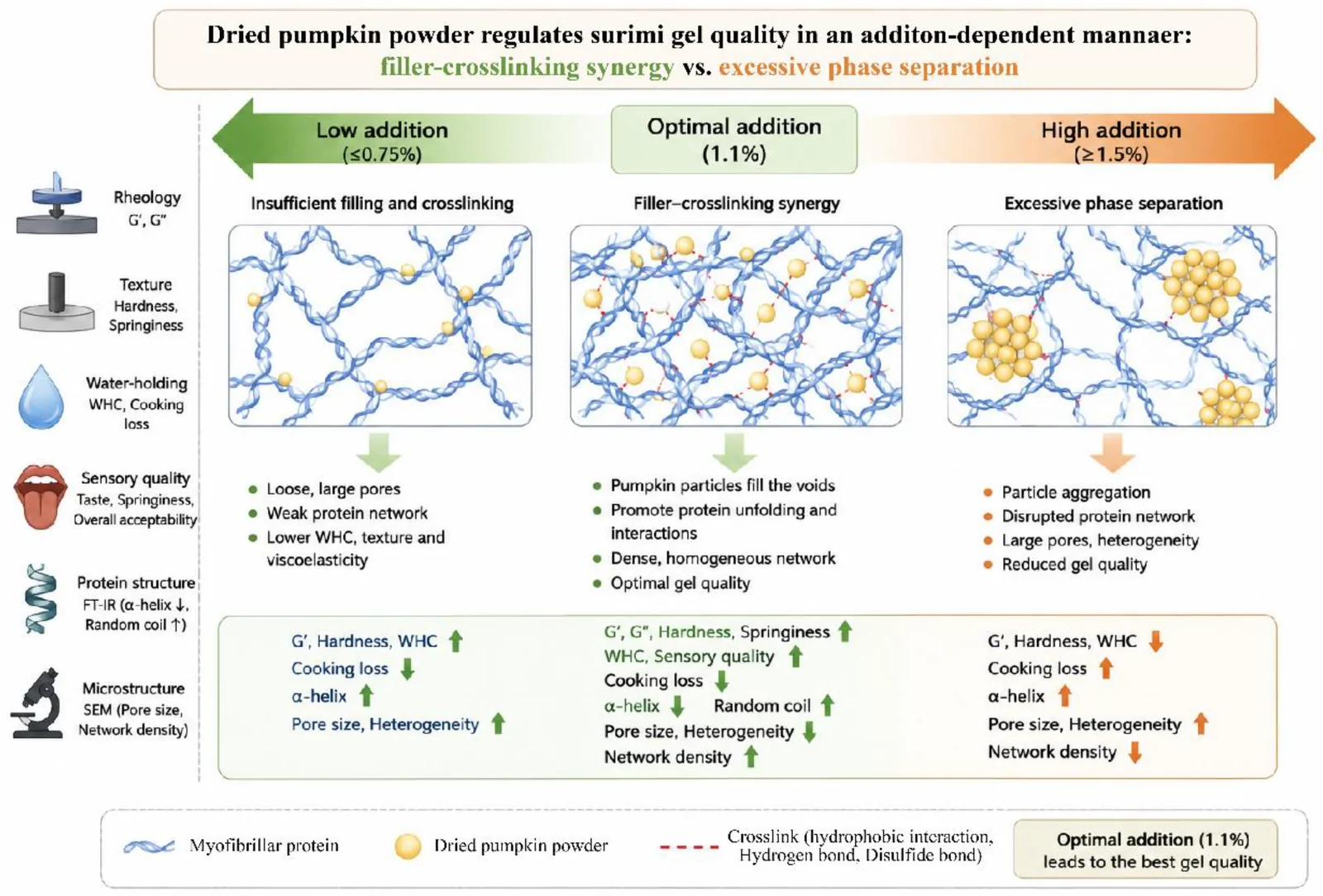
Proposed mechanism illustrating the dose-dependent effects of DPP on the gelation of surimi gel.

Integrating the rheological, textural, water-holding, FT-IR, and microstructural results, a dose-dependent hypothetical model is proposed to explain the effects of DPP on surimi gelation (Figure 8). At the optimal supplementation level of 1.1%, DPP-treated gels exhibited improved textural properties and water-holding capacity, together with marked changes in protein secondary structure and a more compact and homogeneous microstructure. These corresponding changes suggest that the beneficial effects of DPP may be associated with coordinated changes in protein conformation, gel network organization, and water retention. The hydrophilic components of DPP may also contribute to water retention through water binding and physical entrapment within the gel matrix. These three pathways—filler effect, conformational modulation, and water retention—operate in concert to improve texture, water-holding capacity, and sensory quality.

At higher DPP levels (≥1.5%), the gels exhibited larger pores, rougher surfaces, greater structural heterogeneity, and poorer gel properties. These observations suggest that excessive DPP may disrupt the spatial organization of the protein matrix. This biphasic response highlights the critical importance of maintaining an optimal filler—protein balance during gelation.

Collectively, the present results support a proposed structure–conformation–network–function framework in which DPP-induced changes in protein secondary structure, gel microstructure, and water retention are associated with the resulting gel properties. The optimal addition level of 1.1% achieves the most favorable balance among these interrelated factors, providing a theoretical basis for the rational design of DPP-supplemented surimi products.

## 4. Conclusions

This study systematically investigated the effects of DPP on the gelation behavior, physicochemical properties, protein secondary structure, and microstructure of hairtail (*Trichiurus haumela*) surimi gels. Among the tested concentrations, 1.1% DPP was identified as the optimal level, significantly improving gel hardness, springiness, WHC, and sensory quality. FT-IR analysis indicated that moderate DPP supplementation was associated with protein conformational rearrangement, as evidenced by a marked decrease in α-helix content and a corresponding increase in random coil content, thereby facilitating protein aggregation and cross-linking during thermal gelation. SEM observations further showed the formation of a denser and more homogeneous gel network. These molecular and microstructural modifications were consistent with the enhanced rheological and textural properties of the surimi gels. Conversely, excessive pumpkin powder addition (≥1.5%) was associated with greater structural heterogeneity, which disrupted network continuity and compromised gel quality. Collectively, these findings establish a filler–conformation synergistic mechanism by which moderate DPP supplementation improves hairtail surimi gel quality through the coordination of protein structural rearrangement and network consolidation. This study not only provides a scientific basis for the high-value utilization of pumpkin powder as a natural functional ingredient in aquatic food processing but also offers practical guidance for the development of fiber-enriched, high-quality surimi products with improved textural and sensory attributes.

## Figures and Tables

**Table 1:** Effect of DPP addition on the microstructure of surimi gel.

Mass Fraction (%)	0	0.4	0.75	1.1	1.5	1.85
Area (μm^2^)	0.08	0.08	0.08	0.08	0.08	0.08
Perimeter (μm)	9.18	8.98	8.92	8.39	9.66	9.00

## Data Availability

The original contributions presented in this study are included in the article. Further inquiries can be directed to the corresponding author.
